# Promoting Green Product Development Performance via Leader Green Transformationality and Employee Green Self-Efficacy: The Moderating Role of Environmental Regulation

**DOI:** 10.3390/ijerph17186678

**Published:** 2020-09-14

**Authors:** Wengang Zhang, Baiqing Sun, Feng Xu

**Affiliations:** 1 School of Management, Harbin Institute of Technology, Harbin 150001, China; 15B310010@hit.edu.cn (W.Z.); baiqingsun@hit.edu.cn (B.S.); 2 School of Humanities, Social Sciences & Law, Harbin Institute of Technology, Harbin 150001, China

**Keywords:** green transformational leadership, green self-efficacy, environmental regulation, green product development performance

## Abstract

By integrating internal green self-efficacy and external environmental regulation, this research investigates the relationship between green transformational leadership and green product development performance. Taking 23 new energy vehicle enterprises in China as samples, we collected 298 valid questionnaires and verified the hypotheses through structural equation modeling. The results show that both green transformational leadership and green self-efficacy can promote green product development performance; green self-efficacy mediates the positive relationship between green transformational leadership and green product development performance, while environmental regulation positively moderates the mediating effect of green self-efficacy. Furthermore, environmental regulation and green self-efficacy interact to promote green product development performance. Our research provides a new perspective to understand how green transformational leadership is related to green product development performance and how this relationship is molded by contextual antecedents. Enterprises need to comprehensively consider the green influence of transformational leadership, green driving of employees themselves, and green linkage among organizations (macro policy guidance, passive market incentives, and self-issued actions) to improve green product development performance. Limitations and future scope are discussed.

## 1. Introduction

The cause of environmental crisis lies in the rapid evolution of industrialization, and the Chinese government is exploring a new model of harmonious coexistence with nature [1]. Along with the prevalence of environmentalism in the world, consumers pay attention to the impact of consumption and production behavior patterns on the environment, and they encourage environmental action and green consumption [2,3]. Based on the concern for the shared responsibility of the environment, politicians, enterprises, and academia focus on the development of green products [4,5,6].

Green products refer to products with less environmental impact, less harm to human health, formed or partially formed from recyclable components, manufactured in a more energy-efficient manner, or supplied to the market in less packaging [6]. Compared with traditional product development, green product development pays more attention to environmental issues, product life cycle, citation design, and the impact of the entire supply chain on the social environment [7], which plays an important role in enterprises’ response to environmental challenges [8]. Likewise, the widespread acceptance of green products has significantly improved the profitability of corporate green management [9]. Research shows that more and more consumers are willing to pay higher prices for environmentallyfriendly products [10]. Therefore, the development of environmentally friendly products by enterprises can not only satisfy consumers’ growing environmental awareness, but also obtain higher performance from them to maintain competitiveness.

Leaders are considered the key antecedent of workplace innovation performance [11,12], which means that they need to provide contextual support for employee innovation [13]. The research on leadership style is constantly emerging [14], which explores the role of value-oriented leadership as the effective supplement of positive leadership (such as transformational leadership), reflecting its incremental validity, but failing to solve the problem of potential structural redundancy [15]. Additionally, corporate scandals and unethical behaviors have aroused scholars’ doubts about the basic idea of the leadership role [16]. Haque et al. emphasized a normative method of responsible leadership to make up for the lack of responsibility concept in leadership style [17]. Actually, as a dominant leadership theory, transformational leadership focuses on the positive outcomes of leader behavior and interpersonal dynamics [18]. For example, transformational leadership is conducive to improving environmental performance in manufacturing [19]. Similarly, the environmental characteristics of managers are important sources of promoting sustainable practices of enterprises [20]. So, how to reflect not only the value orientation, but also the social responsibility of transformational leadership? We introduce the concept of green transformational leadership.

Green transformational leadership refers to the behavior of leaders to motivate followers to achieve environmental goals, and to motivate followers to exceed the expected level of environmental performance [21,22]. As a promising leadership style, green transformational leadership can provide an inspiring green vision and motivate followers to actively complete environmental goals or tasks, thereby enhancing the company’s green image and possibly bringing green opportunities [23]. As a consequence, green transformational leadership can integrate the concept of environmental management into the product development process, which not only enhances the corporate social responsibility for environmental protection and contributes to sustainable development, but also provides the public with differentiated green product choices [21,23]. Previous studies have found positive links between green transformational leadership and green product development performance in Taiwan manufacturing [21], and we consider the positive impact between the two factors in a broader cross-cultural field (e.g., China mainland).

The development and maintenance of an environmental management system depends on the internal ability of the organization [24,25]. Leaders understand, predict, and control how employees interact to achieve environmental goals [22,26], thereby enhancing corporate green innovation and green performance, especially in a society with high power distance [23,27].Different from focusing on external rewards and leader–employee reciprocity, green transformational leadership, as a kind of pro-environment leadership, follows higher-level self reinforcement and emphasizes that self-regulation is the main driving force for cultivating employees’ specific behaviors [28].

According to social cognitive theory, self-efficacy refers to the belief in one’s ability to execute and organize the action process [29]. Self-efficacy has the attribute of experiential learning, which is easy to be influenced by different leadership styles in the process of subordinates’ work [29,30]. Therefore, when green transformational leadership emerges, it may have a “trickle-down effect”. Similarly, the psychological characteristics of employees can be used to explain corporate performance [31]. Having a high degree of self-confidence makes people more likely to initiate actions, pursue actions, and persevere because they are confident that they can handle what they want or need to do [6,32]. Green self-efficacy refers to the belief in the ability to implement and organize the process of achieving environmental goals [29,32]. Since efficacy beliefs nourish internal motivation by enhancing the perception of self-ability [29,30], green self-efficacy may also reflect intrinsic motivation in environmental activities [6,32].

Previous studies have discussed the antecedents and consequences of green self-efficacy [6], but the specific mediating process of green self-efficacy needs further study. Therefore, this study suggests that the green self-efficacy of employees may be a series item, bridging the conduction route through which green transformational leaders influence green product development performance.

Furthermore, the implementation of environmental management measures by the government, consumers, and other stakeholders has increased the pressure on corporate research and development (R&D) [19,33,34]. Scholars have called on companies to rely on intangible resources to solve complex environmental sustainability problems [35,36]. Current studies have explored the avoidance behavior of innovative work under the constraint of environmental regulation [37,38], ignoring the positive impact of environmental regulation on environmental-related performance. Actually, companies rely on unexpected events in the institutional environment [39], which means that changes in external conditions may lead to production transformation, creative response, and induced innovation [40]. For example, the rapid development of transportation system makes China a country on wheels. In response to climate change and the greenhouse effect, the Chinese government encourages enterprises to pursue considerable environmental performance by setting lower carbon emission standards, developing new energy vehicles, and introducing electric vehicle production lines such as Tesla.

Although the external variables such as environmental regulations and stakeholder pressure are the most frequently targeted variables [39], there is no overall view to explain how environmental regulation, as an environmental factor, complements the green innovative behavior of enterprises, which has a narrow focus that limits the theoretical integrity [37]. Therefore, it is necessary to explore the impact of environmental regulation on green product development performance in China.

**Figure 1:**
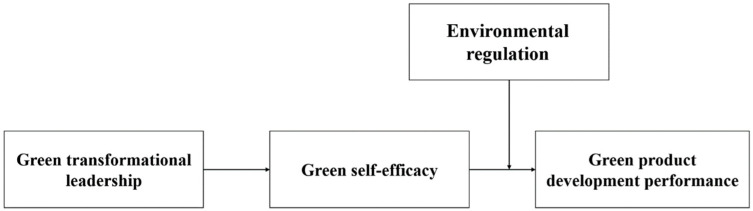
The hypothetical framework.

This research has three contributions: (1) Based on the Chinese context and previous studies by Western scholars, this study aims to demonstrate whether there is regional heterogeneity in the impact of green transformational leadership on green product development performance, and it responds to the proposition of Chinese cultural characteristics [41]. (2) Drawing upon social cognitive theory, this article uses green self-efficacy as a mediated variable to explore the internal endogenous relationships of green innovative activities. As a participant in important decisions, green transformational leadership plays a key role in the sustainable development of the organization. (3) We examine the moderating effect of the intensity of external environmental regulation on corporate green innovation, which is a rare organic combination but worthy of exploring in-depth, and it can also provide a reference for other emerging economies. The hypothetical framework is shown in Figure 1.

## 2. TheoreticalDevelopment and Hypothesis Presentation

### 2.1. Green Transformational Leadership and Green Product Development Performance

The resource-based view indicates that the leader is one of the key resources of the organizational environmental management [19,33]. When the leader makes decisions and takes actions that are conducive to the environment in the organization, he/she will set an example for his/her subordinates [42,43]. Green transformational leadership is the product of transformational leadership in the field of environmental protection [33]. According to Chen and Chang, green transformational leaders take a series of actions to motivate their subordinates to meet environmental protection requirements and encourage them to thrive and surpass the requirements of environmental performance as far as possible [21], which is consistent with the culturally recognized transformational leadership theory discussed by Muralidaran and Pathak [44]; that is, green transformational leadership meets the needs of social green development.

Due to the universality of green development, enterprises seek to transform the production mode to carry out green innovation, and they obtain profits through green product development performance [45,46], which refers to “the development performance of products that have less of an impact on the environment, are less detrimental to human health, are formed or part-formed from recycled components, are manufactured in a more energy-conservative way, or are supplied to the market with less packaging” [21].

Green transformational leadership, on the basis of change orientation, tends to pursue environmental goals, which is similar to responsible leadership, that is, on the basis of interest linkage, more emphasis on social responsibility [16,17]. Therefore, once the enterprise’s green product development involves energy conservation, pollution prevention, and other issues [21], green transformational leadership can provide environmental support for employees, clarify an environmental vision and set environmental expectations, stimulate followers’ awareness of green products, and motivate employees to consider green product development issues from the perspective of environmental sustainability [15,21,22]. Previous studies have shown that green transformational leadership helps formulate green development strategies and promote companies to move toward the goal of green development [21,22,23], thereby promoting green product development performance [21]. Thus,

**Hypothesis 1** **(H1).** *Green transformational leadership can promote green product development performance*.

### 2.2. Green Transformational Leadership and Green Self-Efficacy

The leader’s pro-environmental behavior sets the pace for subordinates [47,48]; accordingly, employees’ psychological motivation can be affected by value-oriented leadership practice [17].

Green transformational leaders, as practitioners of environmental responsibility, can provide sufficient reference and ideal points for subordinates’ pro-environmental behaviors, help them believe that they can successfully overcome current challenges, stimulate their desire to improve environmental protection, influence their behavior, and successfully engage in work related to environmental tasks [21,47].

According to social cognitive theory, employees’ self-confidence in self-efficacy can improve their activity participation [49], which is consistent with transformational leadership theory [47]. Transformational leadership can promote the role’s self-efficacy, because he/she can improve employees’ abilities by coaching their skills [50]. Green self-efficacy can activate individuals’ environmental beliefs and attitudes and then adopt pro-environmental behaviors [32]. Steg pointed out that there is a significant correlation between managers’ environmental beliefs and attitudes and green self-efficacy [51]. Employees’ green self-efficacy can be cultivated by green transformational leadership, which sets feasible environmental goals, clarifies environmental standards, develops environmental culture, and links personal behavior with results [47,49]. For example, a sample survey of 262 employees in the electronics industry in Taiwan shows that there is a positive correlation between green transformational leadership and green self-efficacy [47]. In the same light, we suggest that employees’ confidence to achieve environmental performance can be inspired by green transformational leadership, which embodies a kind of environmental responsibility [22], employees’ green self-efficacy is imperceptibly affected in the top–down communication [48,49], especially in mainland China, which attaches importance to environmental protection.

**Hypothesis 2** **(H2).** *Green transformational leadership can promote employees’ green self-efficacy*.

### 2.3. Green Self-Efficacy and Green Product Development Performance

According to social cognitive theory, self-efficacy is the key to explain the process of driving and regulating behavior [48,52]. Individuals with high self-efficacy may have higher performance and higher commitment to focused on tasks and tolerate failure [46]. Historical studies have shown that self-efficacy is positively correlated with performance [53,54]. Similarly, green self-efficacy reflects an individual’s judgment on his ability to organize and implement the action plan needed to achieve expected environmental performance in anenvironment-driven context [32]. Individuals with higher green self-efficacy have a higher belief in creating and developing new environmental protective products because of the following reasons. (a) Employees care about the compatibility of their long-term career planning with the sustainable development of the organization [23]. If the goal of the organization is to strive to improve green product development performance, in order to fulfill the commitment to environmental goals [21,48], individuals with higher green self-efficacy may achieve higher green performance level through investment and persistence, positive thinking, and self-regulation [32,47]. (b) Individuals with higher green self-efficacy pay attention to the attractiveness of work itself, and they consider environmental issues and corporate social responsibility [32]. In order to find the balance between environment and performance, they will create better job performance by developing more new products; otherwise, they will feel anxiety and uncertainty [55].

**Hypothesis 3** **(H3).** *Green self-efficacy can promote green product development performance*.

### 2.4. Green Self-Efficacy as a Linking Pin between Green Transformational Leadership and Green Product Development Performance

In addition, self-efficacy is related to goal setting, positive feedback, and self-motivation to improve cognitive flexibility, which is conducive to transformational leadership cultivating employees’ self-efficacy [46,50]. From the perspective of stakeholders, responsibility transcends economic interests. Green transformational leadership and green product development performance respectively reflect the environmental commitment of managers and enterprises [21], while employees quickly absorb their superiors’ experience on environmental issues through excellent green self-efficacy and are more likely to achieve higher environmental performance [53]. Although the evidence of green self-efficacy as a potential intermediary in the relationship between green transformational leadership and green product development performance is limited, social cognitive theory is used as a bond to bridge this pathway [47,48].

Combining with the above hypotheses “H1–H3”, we propose that green self-efficacy is a potential linking pin for the internal relevance of the hypothesis H1. Thus,

**Hypothesis 4** **(H4).** *Green self-efficacy mediates the positive relationship between green transformational leadership and green product development performance.*

### 2.5. Two-Way Interaction: Environmental Regulation Moderates the Relationship of “Green Self-Efficacy—Green Product Development Performance”

Cerne et al. argued that whether an individual is innovative at work may depend on the existing criteria for success and failure in the work environment [56]. Environmental regulations realize the process of environmental protection in the process of economic activities via administrative coercion, market incentive, and industry commitment [41]. Administrative coercion makes companies rely on unexpected events in the institutional environment, and they must maintain social norms and rules through legal actions [40]. Strict supervision has created a high-blooded market demand for environmental products [39,41], and the popular consumer demand in the market requires thatcompanies meet consumers’ growing environmental awareness through green production [57]. Voluntary environmental supervision can effectively improve the environmental protection willingness of enterprises and then promote the green innovation output of enterprises [58]. In this case, higher environmental regulations increase the sense of pressure and mission of stakeholders [39,40], and individuals with higher green self-efficacy may use these conditions to create opportunities and promote green product development performance [47,49]. Especially in the context of China’s national conditions, social relationship characteristics are more important than structural characteristics [59]. The closer the cooperation between the organization and the government, the more conducive to obtaining information resources related to environmental regulation and gaining first-mover advantage [41,58]. In order to accomplish the commitment to environmental goals and achieve the balance between environment and performance [60], individuals with higher green-efficacy are likely to use the heterogeneous resources at hand to develop and transform green products to attain higher performance, which is conducive to their own enterprises to formulate green standards, establish potential barriers to entry, and gain a foothold for environmental-oriented competition [37,47].

Previous studies considered environmental regulation as a leading factor to influence the process of economic activities, or regarded environmental regulations as a result to explore the pros and cons of its antecedents, which is not conducive to the completeness of the theory [37,57]. From a macro perspective, environmental regulation, as a focus, inevitably become an interference item in the production and operation of enterprises. Since the essence of human behavior is a function of the continuous multi-directional interaction process between the individual and the environment [61,62], we thus propose environmental regulation as a contextual factor that interacts with individual’s green self-efficacy to influence the innovation results of the enterprise.

**Hypothesis 5** **(H5).** *Environmental regulation moderates the relationship between green self-efficacy and green product development performance, such that this relationship is strengthened when environmental regulation is high rather than low.*

### 2.6. A Moderated Mediation Model

Environmental resources are regarded as a “tangible hand”, which has become an important reason for enterprises to fulfill their environmental responsibilities [63]. Since the “Beautiful China” plan was put forward, China has adopted the most stringent new environmental protection law in history [41], which has made more companies turn their attention to environmental behavior [39]. Green transformational leadership can focus more on pro-environmental behaviors, improve environmental performance, and meet the needs of social green development [21,22]. In Hypothesis 2, we propose that green self-efficacy mediates the positive effect of green transformational leadership on green product development performance. When external policy conditions are binding on the original creators and recipients, the imitators of technical knowledge will weigh the policies to ensure the sustainable development of green economy [64]. With the strengthening of environmental regulations, enterprises will gradually turn to green innovation activities to promote the development of green economy by selectively absorbing green knowledge and technology, developing more green products [65]. Therefore, environmental regulation may moderate the mediated path of Hypothesis 4.

**Hypothesis 6** **(H6).** *Environmental regulation moderates the mediated path in which green transformational leadership affects green product development performance viagreen self-efficacy, such that this linkage is strengthened when environmental regulation is higher*.

## 3. Method

### 3.1. Participants and Procedures

Accompanying the implementation of the “Made in China 2025” strategy, China’s automotive market segmentation scale has reached 1.2 trillion CNY. For example, as the trendsetter in new energy vehicles, the BYD Group has developed electric buses, which are widely used in Europe (such as the UK) and other regions, with a market value of over 263 billion CNY. The explosive growth of the market scale of China’s new energy vehicle companies benefits from the following factors. (1) The Chinese government has given strong support to key industries such as new energy vehicles in terms of policies and funds. (2) Due to the increasingly stringent environmental supervision and the simultaneous improvement of consumers’ green concepts, the automotive industry, as a representative of the manufacturing, is facing the pains of transformation and must develop environmentallyfriendly products such as new energy vehicles to meet environmental challenges. Therefore, we choose China’s new energy vehicle enterprises as the object.

We use a questionnaire survey to investigate new energy vehicle enterprises in six regions including Liaoning, Jilin, Guangdong, Shanghai, Beijing, and Chongqing. These regions have a deep foundation of industrialization and attach importance to environmental issues, which is in line with our research. The measurement scale of the study is derived from mature questionnaires at home and abroad, and two management doctoral students who are familiar with Chinese and English have translated and back-translated the English scale. A bilingual management professor found that the Chinese and English versions of the scale have strong comparability. In addition, considering the influence of cultural background factors, before the questionnaire survey, we interviewed the relevant management personnel of the cooperative enterprise and raised structural questions: Do you think that the enterprise needs environment-oriented transformational leadership to improve the green product development performance? Subsequently, we further adjusted and revised the questionnaire based on the interview results to adapt to China’s context.

Formal surveys were conducted mainly through email and on-site distribution, which were supported by local industrial associations and project partners. Our sample contains data from multiple sources, covering multiple sectors, such as R&D, production, sales, and human resource management, reflecting the heterogeneity of the sample. Green innovation is the core of all business models we choose. For example, a sample company, which is committed to the research and development of pure electric vehicles, extended range electric vehicles, and even hydrogen-powered vehicles, improves green product development performance through independent intelligent processing technology to meet the growing environmental protective demand. The subjects of this study are department managers (such as R&D managers), project leaders, and team members of China’s new energy vehicle industry (including wholly foreign-owned enterprises, state-owned enterprises, and private enterprises). More than 70% of employees are engaged in green innovation, research, and technology development. Therefore, these respondents are suitable for us to observe their green innovative behavior. We surveyed 400 members of 23 companies, and 400 questionnaires were distributed to the target units; of these, 298 valid questionnaires were returned, with an effective response rate of 74.5%. All items were scored with the 7-point Likert scale.

The formal and informal exchanges between invitees within the department are very active. The supervisor may understand the motivation and behavior of subordinates and invite them to complete the questionnaire, which is conducive to avoiding the selective deviation of samples [22]. In addition, the investigator eliminates the social expectation bias by providing the invitees with a confidential survey specification and asking them to sign a survey declaration that guarantees honest answers [47]. In order to reduce the potential common method deviation, we adopt the supervisor–subordinate matching method for paired sampling. Specifically, we require all randomly selected automobile enterprises to provide performance-related green product development key projects; subordinates evaluate green transformational leadership, employees evaluate their green self-efficacy, supervisors evaluate green product development performance, and all members evaluate environmental regulations.

In order to test the non-response bias, we divided the sample into two parts based on the experience of Frazier et al. [66] and compared the samples in terms of enterprise scale, enterprise ownership, and salary level. Statistical analysis of the data set shows that the average age of all employees was 31.67 years (standard deviation = 6.19). Among them, males accounted for 75%, and the average tenure of subordinates and corresponding supervisors was 5.18 years (standard deviation = 2.72). Moreover, 86% of managers were male, with an average age of 38.46 years (standard deviation = 5.51) and an average tenure of 7.33 years (standard deviation = 3.09). A paired t-test showed that there was no significant difference between the two parts tested (*p* > 0.1).

### 3.2. Measurement

#### 3.2.1. Green Transformational Leadership

This study uses the scale revised by Chen and Chang [21], which is derived from Podsakoff et al. [67], to measure employees’ assessment of green transformational leadership of their superiors. The scale contains six items, one sample project, such as “the leader of my green innovation project encourages us to achieve environmental goals”. Cronbach’s α coefficient was 0.94.

#### 3.2.2. Green Self-Efficacy

Green self-efficacy was measured by a five-item scale developed by Guo et al. [32] in this study. A sample is, “I think I can find creative solutions to environmental problems”. Cronbach’s α coefficient was 0.88.

#### 3.2.3. Green Product Development Performance

This research measures green product development performance based on the five scales compiled by Zhou et al. [33]. A sample is, “Green product development projects can achieve their environmental goals in terms of green product development”. Cronbach’s α coefficient was 0.91.

#### 3.2.4. Environmental Regulation

This study measures environmental regulation based on 9 scales compiled by Huo et al. [41]. A sample is, “The department of environmental supervision of the enterprise has strong independence and authority “. Cronbach’s α coefficient was 0.83.

### 3.3. Control Variables

The result of the organizational green behavior is affected by demographic variables [68]; for example, the size and ownership of enterprises will affect the acquisition of corporate resources, which in turn affect green innovation [39]. Therefore, in addition to the size and ownership of enterprises, a total of 6 variables including age, gender, education level, and dual tenure are controlled.

## 4. Results

Table 1 shows the descriptive statistics of the research model structure. Based on software LISREL 8.52 (Scientific Software International Inc., IL, USA), we analyzed the data of multi-item indexes, which may improve the reliability of measurement [69]. The Cronbach coefficient is used to evaluate the reliability of each structure, and the Cronbach’s alpha coefficient ranges from 0.83 to 0.94. According to the standard, once the coefficient exceeds 0.5, the effectiveness of convergence can be reflected [70].

The results in Table 2 exhibit that all the average varianceextracted (AVE) estimates are higher than 0.5. According to previous experience, once the estimated value of AVE exceeds the critical value of 0.5, the validity of the structural measurement is supported [70]. Accordingly, the convergence validity is verified in all measurement scales. The discriminant validity among constructs is evaluated by measuring the square root of AVE for each construct and comparing the values with the construct correlation of each construct [71,72]. As shown in Table 2, in each construct, the correlation coefficient is less than the square root of AVE estimation; thus, our results demonstrate that convergence validity is verified in all measurement scales.

Since our model construction has specific and externally verifiable characteristics, and the respondents are all experienced, the potential common method variance (CMV) bias is resolved. In addition, we assessed the risk of CMV bias by using the Harman single factor test method to conduct an exploratory factor analysis of all the items in the scale used in the study [73]. The results demonstrate that the first principal component explained 18.95% of the variance, indicating that there is not a single factor that can explain most of the variance; thus, CMV is not likely to be a significant concern [74].

We also test the hypotheses model in this study by using structural equation model (SEM) [75], which provides an optimal balance between statistical power and type I error rates [76], especially when testing the mediating effect of the model [77]. Moreover, the tolerance of SEM itself allows explicit modeling measurement errors to reduce the bias caused by parameter estimation [78,79], which is an incomparable advantage of multiple regression [75,80].

**Figure 2:**
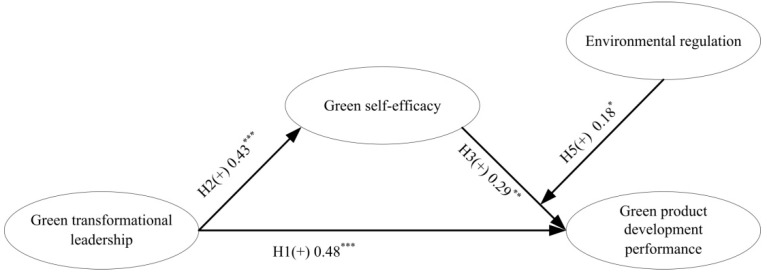
Modelestimationresults. * *p* < 0.05, ** *p* < 0.01, *** *p* < 0.001.

When the mediating effect of the model is related to the underlying structure, as an important tool, SEM provides an analysis strategy for this internal path [81,82,83]. Table 3 and Figure 2 show the path coefficients between variables. In the first step, we used the control variables (age, gender, education, dual tenure, organizational size, and ownership type) as a separate block input to examine their impact on the model. Hierarchical regression analysis showed that age (β = −0.06, *p* > 0.1), gender (β = −0.13, *p* > 0.1), education level (β = 0.17, *p* < 0.05), dual tenure (β = −0.02, *p* > 0.1), organization size (β = −0.05, *p* > 0.1), and ownership type (β = 0.11, *p* > 0.1). These results show that all control variables have no significant correlation with the degree of green product development performance. On the whole, the sum of control variables accounted for 5.6% of the variance of green product development performance (R^2^ = 0.056, *p* > 0.10). Green transformation leadership was positively correlated with green product development performance (0.46; *p* < 0.001).

We further test the mediating effect of green self-efficacy on the relationship of green transformation leadership → green product development performance. According to the criteria of previous studies (the independent variable is significant to the dependent and the mediating variable, the mediating variable is significant to the dependent variable, and the main effect is completely/partially mediated), we propose the following four conditions:

(1) Green transformation leadership is of great significance to green product development performance;
(2) Green transformation leadership is of great significance to green self-efficacy;
(3) Green self-efficacy is of great significance to the performance of green product development;
(4) When the mediator (green self-efficacy) is added to the green transformation leadership and green product development performance model, the standardization coefficient of the green transformation leader’s path influencing green product development performance may become insignificant (complete mediation), or it may be reduced (partial mediation).

Our results show that χ^2^/DF = 2.128, TLI = 0.918, CFI 0.925, and RMSEA = 0.07, describing that the sample data fit the hypothetical model well. The results of H1, H2, and H3 also show that green self-efficacy plays a mediated role in the path from green transformation leadership to green product development performance. The standard coefficient of green transformation leadership leading to green self-efficacy (H2) is 0.43 (*p* < 0.001). The standard coefficient of green self-efficacy leads to green product development performance (H3) is 0.29 (*p* < 0.01). After green self-efficacy is added as an intermediary, the standard coefficient of the green transformation leaders’ path to green product development performance is 0.13, which is significant (*p* < 0.05), because the standard coefficient was lower than that without green self-efficacy (0.48, *p* < 0.001). Thus, the results indicate that green self-efficacy partially mediates the relationship between green transformation leadership and green product development performance. Sobel’s [84] test verified the mediation model hypothesis (z = 2.46, *p* < 0.01). Therefore, H1, H2, H3, and H4 are supported.

In H5, we examine the interaction between green self-efficacy and environmental regulation. The results of the moderating effect show that χ^2^/df = 1.85, NNFI = 0.964, TLI = 0.958, CFI = 0.962, and RMSEA = 0.03, indicating that the fitting degree of the model is better than that of the baseline model. Likewise, the results based on identity trust show that the green self-efficacy of the interaction moderated by environment is significant (β = 0.18, *p* < 0.05). We further draw the relationship between green self-efficacy and green product development performance (defined as +1/−1 standard deviation from the average) to more intuitively reflect the nature of this interactive effect [85].

**Figure 3:**
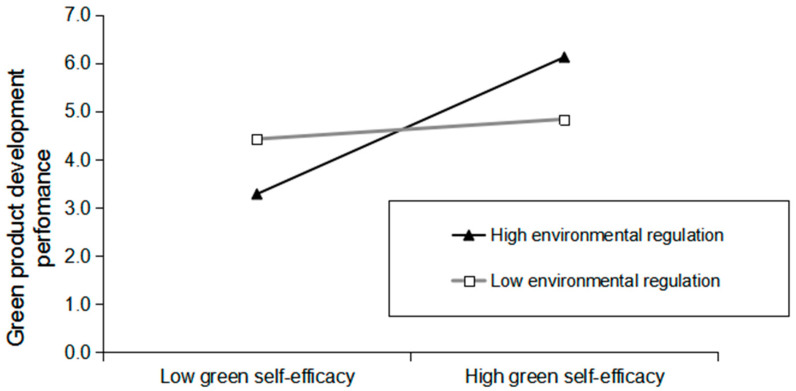
Interaction of green self-efficacy and environmental regulation on green product development performance.

As shown in Figure 3, the simple slope diagram reflects the direction of the interaction effect in the high (M + 1SD) and low (M − 1SD) states of environmental regulation. As anticipated, the positive effect between green self-efficacy and green product development performance is strengthened for employees who evaluate environmental regulation as high, but it is not significant when the perceived environmental regulation is low, confirming H5. Similarly, the moderated mediating effect indicates that the indirect impact of green transformation leadership on green product development performance depends on environmental regulation, such that the indirect effect is strengthened (indirect effect = 0.08) and significant (*p* < 0.05, CI [0.02, 0.15]) when the degree of environmental regulation is higher, rather than lower (indirect effect = 0.02, CI [−0.02, 0.06]); see Table 4. Therefore, Hypothesis 6 is also supported.

## 5. Discussion

In modern society, all circles are widely concerned about how to improve green product development performance through resource integration under the background of environmentalism [21]. Our research examines the relationship of green transformational leadership, green self-efficacy, and green product development performance under the condition of environmental regulation, which not only supports and complements previous literature, but also has practical implications for predicting green innovation in organizations.

### 5.1. Theoretical Contribution

First, we selected the upstream and downstream industrial chains of China’s new energy vehicle enterprises as the object; then, we explored the relationship between green transformational leadership and green product development performance, verified the view of Chen et al. [21], and provided a new perspective for the development of social cognitive theory. Additionally, we analyzed the transmission path of “leadership–performance” influenced by individuals’ green self-efficacy, which partially explains what promotes the organizational green innovation with social cognitive theory.

Second, our results show that environmental constraints in a specific context can not only interact with internal green efficiency to promote green product development performance, but also positively moderate the relationship between the green transformational leadership and green product development performance via green self-efficacy. Therefore, strong environmental supervision can promote environment-oriented green innovation. The environmental characteristics of leaders and the environmental efficiency of employees arekey to promoting the green product development performance of enterprises together under environmental regulation.

Finally, our research advances the development of the literature [22]. Previously, we were mainly rooted in the impact of green transformational leadership on employee green creativity, which is a broad concept because it is a problem worth studying regarding how to transform creativity into achievements and thereby gain specific benefits. Our research finds that green transformational leadership can directly and indirectly promote green product development performance, which strengthens the relationships among “leadership–cognition–creativity–performance” under the constraints of an environmental system, and it is also a beneficial supplement to previous research [21,22].

### 5.2. Practical Value

First, from the perspective of the external environment, modern organizations should pay special attention to the impact of environmental regulation when considering product performance affected by environmental factors, especially in China, which is increasingly focusing on ecological performance. Technology, regulatory promotion, and market pull all force companies to no longer symbolically pursue the legitimacy of production, but rather improve environmental performance through the rational allocation of resources and benign interaction with stakeholders [37,41]. Environmental regulation is the filtering mechanism of technology and the green economy. In this category, enterprises can increase environmental investment and green innovative activities by highlighting technological advantages and introducing novel ideas, thereby enhancing their green product development capabilities.

Second, from the perspective of internal structure, enterprises should consider the influence of leadership on employees’ psychological motivation except for technical factors in the process of developing green product development performance. Green transformational leadership not only directly affects employees’ green self-efficacy, but also directly and indirectly affects the enterprises’ green product development performance. Accordingly, enterprises should pay attention to managers with high moral obligations to social responsibility [16] and provide a learning environment outside the formal workplace to enhance leaders’ sense of responsibility [17,86], thereby promoting the green innovation of enterprises. Furthermore, as stakeholders, employees should have more opportunities to be supported and understood in human resources practice [86]. Enterprises can improve employees’ green self-efficacy through green recruitment, targeted job training (such as hiring tutors), and team building, and they can provide competitive salary plans to improve employees’ organizational commitment to promote green product development. Therefore, companies can focus on environmental-oriented transformational leadership and self-efficacy to form a synergy and obtain green benefits while responding to market demands.

Last but not least, from the perspective of coordinating internal and external situations, companies should spontaneously establish a dynamic sustainability standard according to specific guidelines to cope with the growing pressure of transformation and development. Green innovation has become an effective way to differentiate the management and sustainable development of enterprises [6]. As the most representative new economy in the world, China’s environmental regulation requirements are constantly improved with the increase ofthe development of industrialization. For example, Li et al. pointed out that companies have begun to use ISO14001 certification to achieve sustainable development [39]. Therefore, while pursuing benefits and measures that link environmental assessment mechanisms to personal performance and job promotion, designing and adjusting appropriate ecological innovation systems can be used by enterprises to improve their green product development performance and develop a green economy.

### 5.3. Future Scope

We summarize the limitations of this article and propose three future directions.

First, the experimental design is cross-sectional, which may limit the interpretation of causal chain. Further research can find stability and key nodes in the experiment process through longitudinal tracking investigation.

Second, although we selecteddepartments of R&D, production, sales, and after-sales in the new energy vehicle industry as the object, the research is still rooted in the manufacturing industry. Since there may be differences in the green activities of different industries, we will test the universality of the hypotheses by expanding more industries (such as the hotel industry, banking) and samplesin the future. In addition, it may be an advisable choice to conduct a comparative demonstration of cross-cultural and institutional comparisons between China and the West.

Finally, this research analyzes the impact of green transformational leadership, green self-efficacy, and environmental regulation on green product developmentperformance. In the future, we will develop more variables (such as establishing sequential mediation: leadership–psychological motivations–job characteristics–innovative performance), or construct more parallel paths (for example, the development of two unrelated but similar mediated variables for comparison) to explore the antecedents that affect green product development performance, as well as specific result-oriented case arguments.

## 6. Conclusions

In summary, we investigate the effectiveness of green transformational leadership (as a value-oriented and environmental responsible leadership) on green product development performance in the Chinese context, and we extend social cognitive theory by testing green self-efficacy as a linking pin to bridge the relationship of “leadership–performance”. Additionally, our results show that environmental regulation, as an accidental factor, can not only interact with green self-efficacy, but also moderate the transmission process of green self-efficacy. Our research indicates the antecedents of enterprises’ green behavior and the boundary conditions of the causal linkage. This research echoes the “Beautiful China” plan, which provides a distinctive perspective on how enterprises carry out green innovation and assume social responsibility under the strategic deployment of “China Intelligent Manufacturing”.

## Figures and Tables

**Table 1:** Descriptive statistics of the constructs.

Constructs	No. of Items	Cronbach’s Alpha	Loadings Range	Average Variance Extracted	χ^2^/df	NNFI	CFI	RMSEA
Green transformational leadership	6	0.94	[0.85–0.96]	0.759	1.43	0.943	0.956	0.02
Green self-efficacy	5	0.88	[0.79–0.90]	0.681	1.55	0.918	0.948	0.05
Green product development performance	5	0.91	[0.82–0.95]	0.724	1.79	0.925	0.953	0.04
Environmental regulation	9	0.83	[0.75–0.89]	0.667	2.32	0.902	0.950	0.03

**Table 2:** Measurement model: discriminant validity.

Construct	Green Transformational Leadership	Green Self-Efficacy	Green Product Development Performance	Average Variance Extracted
Green transformational leadership	0.871			0.759
Green self-efficacy	0.696	0.825		0.681
Green product development performance	0.721	0.684	0.851	0.724
Environmental regulation	0.623	0.421	0.703	0.667

Note: Square root of average variance extracted (AVE) for each construct was shown in the diagonal of the correlation matrix.

**Table 3:** Results of path coefficient analysis.

Hypothesis	Description of Path	Path Coefficient	Conclusion
H1	green transformational leadership→green product development performance	0.48 ***	H1(+): supported
H2	green transformational leadership→green self-efficacy	0.43 ***	H2(+): supported
H3	green self-efficacy→green product development performance	0.29 **	H3(+): supported

Notes: χ^2^/df = 1.81, NNFI = 0.96; TLI = 0.96; CFI = 0.96; RMSEA = 0.02. Tests of hypotheses are two-tailed tests; ** *p* < 0.01; *** *p* < 0.001.

**Table 4:** Conditional indirect relationship between green transformational leadership and green product development performance through green self-efficacy at low and high values of environmental regulation.

Environmental Regulation	Conditional Indirect Effect	SE	95% Confidence Interval Lower Limit	95% Confidence Interval Upper Limit
High(M + 1SD)	0.08 *	0.02	0.02	0.15
Low(M − 1SD)	0.02	0.02	−0.02	0.06

Note. *n* = 298. Bootstrap sample size = 10,000; * *p* < 0.05.

## References

[1] Peng J., Chen X., Zou Y., Nie Q. (2020). Environmentally specific transformational leadership and team pro-environmental behaviors: The roles of pro-environmental goal clarity, pro-environmental harmonious passion, and power distance. Hum. Relat..

[2] Lin S.-T., Niu H.-J. (2018). Green consumption: Environmental knowledge, environmental consciousness, social norms, and purchasing behavior. Bus. Strat. Environ..

[3] Souri M.E., Sajjadian F., Sheikh R., Sana S.S. (2018). Grey SERVQUAL method to measure consumers’ attitudes towards green products: A case study of Iranian consumers of LED bulbs. J. Clean. Prod..

[4] Fraccascia L., Giannoccaro I., Albino V. (2018). Green product development: What does the country product space imply?. J. Clean. Prod..

[5] Ilg P. (2018). How to foster green product innovation in an inert sector. J. Innov. Knowl..

[6] Chen Y.-S., Chang T.-W., Lin C.-Y., Lai P.-Y., Wang K.-H. (2016). The influence of proactive green innovation and reactive green innovation on green product development performance: The mediation role of green creativity. Sustainability.

[7] Dey K., Roy S., Saha S. (2018). The impact of strategic inventory and procurement strategies on green product design in a two-period supply chain. Int. J. Prod. Res..

[8] Le Van Q., Viet Nguyen T., Nguyen M.H. (2019). Sustainable development and environmental policy: The engagement of stakeholders in green products in Vietnam. Bus. Strat. Environ..

[9] Chen Y.S., Hung S.T., Wang T.Y., Huang A.F., Liao Y.W. (2017). The influence of excessive product packaging on green brand attachment: The mediation roles of green brand attitude and green brand image. Sustainability.

[10] Li Q., Long R., Chen H. (2018). Differences and influencing factors for Chinese urban resident willingness to pay for green housings: Evidence from five first-tier cities in China. Appl. Energy.

[11] Afsar B., Masood M. (2017). Transformational leadership, creative self-efficacy, trust in supervisor, uncertainty avoidance, and innovative work behavior of nurses. J. Appl. Behav. Sci..

[12] Yang H., Yang J. (2018). The effects of transformational leadership, competitive intensity and technological innovation on performance. Technol. Anal. Strateg. Manag..

[13] Schuh S.C., Zhang X., Morgeson F.P., Tian P., van Dick R. (2017). Are you really doing good things in your boss’s eyes? Interactive effects of employee innovative work behavior and leader-member exchange on supervisory performance ratings. Hum. Resour. Manag..

[14] Ng T.W.H., Feldman D.C. (2012). Idiosyncratic Deals and Voice Behavior. J. Manag..

[15] Hoch J.E., Bommer W.H., Dulebohn J.H., Wu D. (2016). Do ethical, authentic, and servant leadership explain variance above and beyond transformational leadership? A meta-analysis. J. Manag..

[16] Haque A., Fernando M., Caputi P. (2017). The relationship between responsible leadership and organisational commitment and the mediating effect of employee turnover intentions: An empirical study with australian employees. J. Bus. Ethics.

[17] Haque A., Fernando M., Caputi P. (2018). Responsible leadership, affective commitment and intention to quit: An individual level analysis. Leadersh. Organ. Dev. J..

[18] Kim S., Shin M. (2017). Transformational leadership behaviors, the empowering process, and organizational commitment: Investigating the moderating role of organizational structure in Korea. Int. J. Hum. Resour. Manag..

[19] Singh S.K., Giudice M.D., Chierici R., Graziano D. (2020). Green innovation and environmental performance: The role of green transformational leadership and green human resource management. Technol. Forecast. Soc. Chang..

[20] Robertson J.L., Barling J. (2017). Contrasting the nature and effects of environmentally specific and general transformational leadership. Leadersh. Organ. Dev. J..

[21] Chen Y.-S., Chang C.-H. (2012). The Determinants of green product development performance: Green dynamic capabilities, green transformational leadership, and green creativity. J. Bus. Ethics.

[22] Zhang W., Xu F., Wang X. (2020). How green transformational leadership affects green creativity: Creative process engagement as intermediary bond and green innovation strategy as boundary spanner. Sustainability.

[23] Mittal S., Dhar R.L. (2016). Effect of green transformational leadership on green creativity: A study of tourist hotels. Tour. Manag..

[24] Biscotti A.M., D’Amico E., Monge F. (2018). Do environmental management systems affect the knowledge management process? The impact on the learning evolution and the relevance of organisational context. J. Knowl. Manag..

[25] Dangelico R.M., Pujari D., Pontrandolfo P. (2016). Green product innovation in manufacturing firms: A sustainability-oriented dynamic capability perspective. Bus. Strat. Environ..

[26] Northouse P.G. (2015). Leadership: Theory and Practice.

[27] AlMazrouei H., Zacca R., Bilney C., Antoine G. (2016). Expatriate managers decision- making practices within the UAE: A qualitative study. Int. J. Organ. Anal..

[28] Wang C.-J., Tsai H.-T., Tsai M.-T. (2014). Linking transformational leadership and employee creativity in the hospitality industry: The influences of creative role identity, creative self-efficacy, and job complexity. Tour. Manag..

[29] Bandura A. (1997). Self-Efficacy: The Exercise of Control.

[30] Fuchs C., Sting F., Schlickel M., Alexy O. (2019). The ideator’s bias: How identity-induced self-efficacy drives overestimation in employee-driven process innovation. Acad. Manag. J..

[31] Palmer C., Niemand T., Stöckmann C., Kraus S., Kailer N. (2019). The interplay of entrepreneurial orientation and psychological traits in explaining firm performance. J. Bus. Res..

[32] Guo L.J., Xu Y., Liu G.F., Wang T., Du C.L. (2019). Understanding firm performance on green sustainable practices through managers’ ascribed responsibility and waste management: Green self-efficacy as moderator. Sustainability.

[33] Zhou S., Zhang D., Lyu C., Zhang H. (2018). Does seeing “mind acts upon mind” affect green psychological climate and green product development performance? The role of matching between green transformational leadership and individual green values. Sustainability.

[34] Yu W., Ramanathan R., Nath P. (2017). Environmental pressures and performance: An analysis of the roles of environmental innovation strategy and marketing capability. Technol. Forecast. Soc. Chang..

[35] Dubey R., Gunasekaran A., Ali S.S. (2015). Exploring the relationship between leadership, operational practices, institutional pressures and environmental performance: A framework for green supply chain. Int. J. Prod. Econ..

[36] Singh S.K., El-Kassar A.N. (2019). Role of big data analytics in developing sustainable capabilities. J. Clean. Prod..

[37] Liao Y.-C., Tsai K.-H. (2018). Innovation intensity, creativity enhancement, and eco-innovation strategy: The roles of customer demand and environmental regulation. Bus. Strat. Environ..

[38] Teeter P., Sandberg J. (2016). Constraining or enabling green capability development? How policy uncertainty affects organizational responses to flexible environmental regulations. Br. J. Manag..

[39] Li D., Tang F., Jiang J. (2019). Does environmental management system foster corporate green innovation? The moderating effect of environmental regulation. Technol. Anal. Strateg..

[40] Mi Z.F., Zeng G., Xin X.R., Shang Y.M., Hai J.J. (2018). The extension of the Porter hypothesis: Can the role of environmental regulation on economic development be affected by other dimensional regulations?. J. Clean. Prod..

[41] Huo W., Li X., Zheng M., Liu Y., Yan J. (2020). Commitment to human resource management of the top management team for green creativity. Sustainability.

[42] Wang X., Zhou K., Liu W. (2018). Value Congruence: A study of green transformational leadership and employee green behavior. Front. Psychol..

[43] Tuan L.T. (2019). Catalyzing employee Ocbe in tour companies: The role of environmentally specific charismatic leadership and organizational justice for pro-environmental behaviors. J. Hosp. Tour. Res..

[44] Muralidharan E., Pathak S. (2018). Sustainability, transformational leadership and social entrepreneurship. Sustainability.

[45] Gabler C.B., Richey R.G., Rapp A. (2015). Developing an eco-capability through environmental orientation and organizational innovativeness. Ind. Mark. Manag..

[46] Chen T., Li F., Leung K. (2016). When does supervisor support encourage innovative behavior? Opposite moderating effects of general self-efficacy and internal locus of control. Pers. Psychol..

[47] Chen Y.-S., Chang C.-H., Lin Y.-H. (2014). Green transformational leadership and green performance: The mediation effects of green mindfulness and green self-efficacy. Sustainability.

[48] Bandura A. (1986). Social Foundations of Thought and Action: A Social Cognitive Theory.

[49] Chen Y.-S., Chang C.-H., Yeh S.-L., Cheng H.-I. (2014). Green shared vision and green creativity: The mediation roles of green mindfulness and green self-efficacy. Qual. Quan..

[50] Azim M.T., Fan L., Uddin M.A., Abdul Kader Jilani M.M., Begum S. (2019). Linking transformational leadership with employees’ engagement in the creative process. Manag. Res. Rev..

[51] Steg L. (2010). Explaining prosocial intentions: Testing causal relationships in the norm activation model. Br. J. Soc. Psychol..

[52] Beck J.W., Schmidt A.M. (2015). Negative relationships between self-efficacy and performance can be adaptive: The mediating role of resource allocation. J. Manag..

[53] Honicke T., Broadbent J. (2016). The influence of academic self-efficacy on academic performance: A systematic review. Educ. Res. Rev..

[54] Rapp A., Baker T.L., Bachrach D.G., Ogilvie J., Beitelspacher L.S. (2015). Perceived customer show rooming behavior and the effect on retail salesperson self-efficacy and performance. J. Retail..

[55] Xing X.P., Liu T.S., Wang J.H., Shen L., Zhu Y. (2019). Environmental regulation, environmental commitment, sustainability exploration/exploitation innovation, and firm sustainable development. Sustainability.

[56] Cerne M., Hernaus T., Dysvik A., Skerlavaj M. (2017). The role of multilevel synergistic interplay among team mastery climate, knowledge hiding, and job characteristics in stimulating innovative work behavior. Hum. Resour. Manag. J..

[57] Benhong P., Yu T., Guo W. (2018). Can environmental regulations promote corporate environmental responsibility? Evidence from the moderated mediating effect model and an empirical study in china. Sustainability.

[58] Li D., Cao C., Zhang L., Chen X., Ren S., Zhao Y. (2017). Effects of corporate environmental responsibility on financial performance: The moderating role of government regulation and organizational slack. J. Clean. Prod..

[59] Huang J.W., Li Y.H. (2017). Green innovation and performance: The view of organizational capability and social reciprocity. J. Bus. Ethics.

[60] Wang Y., Li Y.X., Ma Z., Song J.B. (2019). The deterrence effect of a penalty for environmental violation. Sustainability.

[61] Wu C.H., Parker S.K., deJong J.P. (2014). Need for cognition as an antecedent of individual innovation behavior. J. Manag..

[62] Zhang W., Xu F., Sun B. (2020). Are open individuals more creative? The interaction effects of leadership factors on creativity. Pers. Indiv. Differ..

[63] Bergman M.M., Bergman Z., Berger L. (2017). An empirical exploration, typology, and definition of corporate sustainability. Sustainability.

[64] Cai W., Zhou X. (2014). On the drivers of eco-innovation: Empirical evidence from China. J. Clean. Prod..

[65] Zhao S., Jiang Y., Wang S. (2019). Innovation stages, knowledge spillover, and green economy development: Moderating role of absorptive capacity and environmental regulation. Environ. Sci. Pollut. Res..

[66] Frazier G.L., Maltz E., Antia K.D., Rindfleisch A. (2009). Distributor sharing of strategis information with suppliers. J. Mark..

[67] Podsakoff P.M., MacKenzie S.B., Moorman R.H., Fetter R. (1990). Transformational leader behaviors and their effects on followers’ trust in leader, satisfaction, and organizational citizenship behaviours. Leadersh. Q..

[68] Kim A., Kim Y., Han K., Jackson S.E., Ployhart R.E. (2017). Multilevel influences on voluntary workplace green behavior: Individual differences, leader behavior, and coworker advocacy. J. Manag..

[69] Neuman W.L. (2000). Social Research Methods: Qualitative and Quantitative Approaches.

[70] Fornell C., Larcker D.F. (1981). Evaluating structural equation models with unobservable and measurement errors. J. Mark. Res..

[71] Tabachnick B.G., Fidell L.S. (2001). Using Multivariate Statistics.

[72] Bollen K.A., Long J.S. (1989). Testing Structural Equation Models.

[73] Podsakoff P.M., Organ D.W. (1986). Self-reports in organizational research: Problems and prospects. J. Manag..

[74] Dubey R., Gunasekaran A., Childe S.J. (2018). Big data analytics capability in supply chain agility: The moderating effect of organizational flexibility. Manag. Decis..

[75] Iacobucci D., Saldanha N., Deng X. (2007). A meditation on mediation: Evidence that structural equations models perform better than regressions. J. Consum. Psychol..

[76] MacKinnon D.P., Lockwood C.M., Hoffman J.M., West S.G., Sheets V. (2002). A comparison of methods to test mediation and other intervening variables effects. Psychol. Methods.

[77] Cheung G.W., Lau R.S. (2008). Testing mediation and suppression effects of latent variables: Bootstrapping with structural equation models. Organ. Res. Methods.

[78] Beltran-Martin I., Roca-Puig V., Escrig-Tena A., Bou-Llusar J.C. (2008). Human resource flexibility as a mediating variable between high performance work systems and performance. J. Manag..

[79] Anderson J.C., Gerbing D.W. (1988). Structural equation modeling in practice: A review and recommended two-step approach. Psychol. Bull..

[80] Bou J.C., Satorra A. (2010). A multi group structural equation approach: A demonstration by testing variation of firm profitability across eu samples. Organ. Res. Methods.

[81] Cepeda Carrión G., Nitzl C., Roldán J.L., Latan H., Noonan R. (2017). Mediation analyses in partial least squares structural equation modeling: Guidelines and empirical examples. Partial Least Squares Path Modeling: Basic Concepts, Methodological Issues and Applications.

[82] Baron R.M., Kenny D.A. (1986). The moderator–mediator variable distinction in social psychological research: Conceptual, strategic, and statistical considerations. J. Pers. Soc. Psychol..

[83] Judd C.M., Kenny D.A. (1981). Process analysis estimating mediation in treatment evaluations. Eval. Rev..

[84] Sobel M.E., Leinhardt S. (1982). Asymptotic confidence intervals for indirect effects in structural equation models. Sociological Methodology.

[85] Aiken L.S., West S.G. (1991). Multiple Regression: Testing and Interpreting Interactions.

[86] Haque A., Fernando M., Caputi P. (2020). How is responsible leadership related to the three-component model of organisational commitment?. Int. J. Prod. Perf. Manag..

